# Physical activity and mobility disability in older adult cancer survivors

**DOI:** 10.1093/jncics/pkaf084

**Published:** 2025-09-10

**Authors:** Justin C Brown, Shengping Yang

**Affiliations:** AdventHealth, Orlando, FL 32804, United States; Pennington Biomedical Research Center, Baton Rouge, LA 70808, United States

## Abstract

**Background:**

Cancer survivors may be more likely to experience accelerated declines in physical function compared to cancer-free controls, but objective data and knowledge of preventive interventions are limited.

**Methods:**

The Lifestyle Interventions and Independence for Elders (LIFE) study was a multicenter, single-blinded, randomized trial conducted at 8 centers across the United States that enrolled 1635 sedentary adults aged 70-89 years and with physical limitations but who could walk 400 m at baseline, of which 371 (22.7%) reported a history of cancer. Participants were randomized in a 1:1 ratio to a health education or physical activity program. The primary endpoint was time to major mobility disability, defined objectively by the inability to walk 400 m in less than 15 minutes.

**Results:**

Cancer history modified the effect of randomized group on major mobility disability (*P* = .006). Among those randomized to the health education program, participants with a history of cancer were 53% more likely to develop major mobility disability compared with participants who did not have a history of cancer (Hazard Ratio (HR) = 1.53; 95% CI = 1.18 to 1.99; *P* = .001). Among participants with a history of cancer, those randomized to the physical activity program were 43% less likely to develop major mobility disability compared with the health education program (HR = 0.57; 95% CI = 0.40 to 0.82; *P* = .003).

**Conclusion:**

In this analysis of a randomized clinical trial, cancer survivors had an increased risk of mobility disability compared with non-cancer controls, and physical activity attenuated this risk.

## Introduction

The prevalence of older adults who survive 5-10 years after cancer continues to increase,^1^ and the primary unmet need of this population relates to physical health.^2^^,^^3^ Adequate physical function is essential for safe and independent living.^4^ Cancer survivors may be more likely to experience accelerated declines in physical function compared to cancer-free controls.^5-7^ Such declines occur irrespective of cancer type and stage of disease and often do not recover after the completion of cancer therapy.^5^^,^^6^ Declines in physical function are associated with an increased risk of mortality in cancer survivors.^8^ Interventions that prevent functional decline may extend the health span of cancer survivors.^9^

The Lifestyle Interventions and Independence for Elders (LIFE) study reported that physical activity statistically significantly reduced the risk of major mobility disability—objectively defined by the inability to walk 400 m—by 18% over a median of 2.6 years compared with health education among 1635 sedentary adults aged 70-89 years.^10^ More than 1 in 5 LIFE study participants self-reported a history of cancer at trial enrollment.^10^ but the specific effect of physical activity on mobility disability in this high-risk cancer survivor population remains uncertain.^11^ This analysis tested the hypothesis that a history of cancer at enrollment modifies the effect of randomized group on major mobility disability risk.

## Methods

### Trial design

The study used a randomized, parallel-group, controlled design conducted at 8 performance sites across the United States that included urban, suburban, and rural communities (University of Florida, Gainesville and Jacksonville, Florida; Northwestern University, Chicago, Illinois; Pennington Biomedical Research Center, Baton Rouge, Louisiana; University of Pittsburgh, Pittsburgh, Pennsylvania; Stanford University, Stanford, California; Tufts University, Boston, Massachusetts; Wake Forest School of Medicine, Winston-Salem, North Carolina; and Yale University, New Haven, Connecticut). The Administrative Coordinating Center was at the University of Florida, and the Data Management, Analysis, and Quality Control Center was at the Wake Forest School of Medicine.

The study followed Good Clinical Practice and the ethical principles in the Declaration of Helsinki. The Institutional Review Board at each performance site approved the protocol and informed consent document. All subjects provided written informed consent. The trial was monitored by an independent Data Safety and Monitoring Board whose members were appointed by the National Institute on Aging (NIA). The study was registered on Clinicaltrials.gov as NCT01072500. Additional details of the trial design have been described.^12^

### Participants

Eligible participants were older adults aged 70-89 years; sedentary (defined as less than 125 minutes per week of moderate-intensity physical activity on the Community Healthy Activities Model Program for Seniors-18 questionnaire)^13^; at high-risk for mobility disability (defined as a Short Physical Performance Battery (SPPB) score less than or equal to 9)^14^; who could walk 400 m in less than 15 minutes without sitting, leaning, or the help of another person or walker; without major cognitive impairment (defined as a Modified Mini-Mental State Examination 1.5 SD below education- and race-specific normative values)^15^; could safely participate in the intervention; and were willing to be randomized. Individuals who reported a history of colon, rectal, prostate, uterine, breast, cervical, thyroid, and oral cancer were eligible if chemotherapy or radiotherapy was completed before enrollment. Other cancer types were eligible if permitted by the study physician at the performance site in consultation with the participant’s physician. Additional details of the inclusion and exclusion criteria have been described.^12^

### Randomization and blinding

Participants were randomized in a 1:1 ratio to a health education program (attention control) or a physical activity program (active intervention) with a secure, web-based data management system that used a permuted block algorithm (with random block lengths).^16^ Randomization was stratified by sex and performance site. Endpoint assessors were blinded to treatment assignment, but participants and the staff who delivered the interventions were not blinded to treatment assignment.

### Health education group

The health education program focused on improving participant knowledge about aspects of successful aging. The health education group attended weekly interactive and didactic workshops that lasted 60-90 minutes during the first 26 weeks and then monthly sessions thereafter. The topics selected were tailored to older adults, such as effectively negotiating the health care system, how to travel safely, preventive services and screenings, where to go for reliable health information and legal and financial issues. The workshops did not include any topics on physical activity. The program included 10 minutes of instructor-led upper extremity stretching or flexibility exercises.

### Physical activity group

The physical activity program focused on walking, strength, balance, and flexibility. Participants were asked to attend 2 center-based visits per week and complete home-based activities 3-4 times per week for the duration of the study. The physical activity sessions were individualized and progressed to 30 minutes of walking daily at moderate intensity towards a goal of 150 minutes per week, 10 minutes of lower extremity strength training using ankle weights, 10 minutes of balance training, and large muscle group flexibility exercises. The program started at a lighter intensity and gradually increased over the first 2-3 weeks. Additional details of the physical activity program have been described.^17^

### Endpoint measures

The endpoint of major mobility disability was defined as the inability to complete a 400-m walk test within 15 minutes without sitting and without the help of another person or walker. This endpoint was selected as an objective indicator of the ability to function independently in the community; for example, participants unable to complete the 400-m walk test in 15 minutes have a very slow gait speed (less than 0.45 m/s), which renders walking capacity in daily life extremely difficult (eg, not fast enough to cross a typical street intersection with timed pedestrian cross signals).^18^^,^^19^ Participants walked 400 m at their usual pace without overexerting themselves, completing 10 laps of a 20-m walking course (40 m/lap). Participants could stop for up to one minute for fatigue or related symptoms. The use of a cane was permitted. Major mobility disability was assessed every 6 months at clinic visits.

When major mobility disability could not be objectively measured because of the inability of the participant to attend the clinic visit and the absence of a suitable walking course at the participant’s home, institution, or hospital, an alternative adjudication of the outcome was based on objective inability to walk 4 m in less than 10 seconds, or self-, proxy-, or medical record-reported inability to walk across a room. If participants met these alternative criteria, they would not be able to complete the 400-m walk within 15 minutes. A panel, blinded to the intervention assignment, adjudicated participants in situations where the 400-m walk could not be performed (eg, the participant was hospitalized or seen at home, where a suitable walk course was unavailable); 14% of events were based on adjudication. The endpoint of persistent mobility disability was defined as 2 consecutive major mobility disability assessments or major mobility disability followed by death.

### Cancer history

At trial enrollment, participants self-reported a medical history of invasive cancer. Participants with a positive cancer history reported the type of cancer using prespecified categories. Participants reported the date of diagnosis and receipt of cancer-directed treatments using prespecified categories (surgery, chemotherapy, radiotherapy, hormonal therapy, and any other treatments). Participants could report more than one type of cancer.

### Other measures

Demographic characteristics, including age, sex, race, and ethnicity, were self-reported at baseline. The SPPB is a brief functional assessment based on a balance test, timed short-distance walk, and repeated chair stands; each assigned a score ranging from 0 to 4, generating a summary score ranging from 0 (worst performers) to 12 (best performers).^14^^,^^20^ Body mass index (BMI) was quantified using staff-measured height and weight. Participants wore a triaxial accelerometer (Actigraph GT3X+, Pensacola, FL, USA) on their waist for 7 consecutive days at baseline and 0.5, 1, and 2 years to quantify moderate-intensity physical activity, calculated using validated cut points.^21^^,^^22^

### Statistical analysis

Baseline characteristics were summarized using means and SD for continuous variables and counts with percentages for categorical variables. Intervention adherence was compared using the Kruskal-Wallis test. Accelerometer-quantified physical activity was analyzed using a mixed model for repeated measures that included group, visit, cancer, group-by-visit, group-by-cancer, visit-by-cancer, and a group-by-visit-by-cancer interaction terms, the baseline physical activity volume, and randomization stratification factors sex and performance site. The adjusted between-group mean difference was quantified as the estimated treatment difference with corresponding 95% CI. The primary endpoint was the time until the first post-randomization occurrence of major mobility disability, and the key secondary endpoint was persistent mobility disability. Participants contributed follow-up time to the analysis until the last definitive assessment (400-m walk or alternative adjudication, as described earlier) for major mobility disability, after which they were censored. For participants who did not have any outcome assessment, 1 hour of follow-up time was assigned because participants had completed the 400-m walk at baseline. At the time this exploratory analysis was designed, the sample size of participants with a history of cancer was known (*n* = 371); conservatively assuming the incidence of mobility disability in participants with a history of cancer was similar to that of the health education group in the overall intention-to-treat analysis, a sample size of 371 provided 80% statistical power to detect a HR of 0.58. The hazard of major mobility disability by randomized group was estimated using a Cox regression model. The model terms included group, cancer history, a group-by-cancer history interaction term, and the randomization stratification factors sex and performance site. Sensitivity analysis was conducted that further adjusted for baseline characteristics that differed between participants with a history of cancer and those who did not report a history of cancer, including age, race, BMI, and physical activity volume. Effect modification by cancer history was examined using the nested likelihood ratio test. Adverse events were estimated using generalized linear models. No adjustments were made for multiple testing. Data were analyzed using Stata v.17.1 (College Station, TX).

## Results

From February 2010 to December 2011, 1635 participants were eligible and randomized,^23^ of which 371 (22.7%) reported a history of cancer (Table 1; Figure S1). The most common types of cancer included breast (*n* = 114), prostate (*n* = 94), and colorectal (*n* = 38), diagnosed a mean (SD) of 11.9 (9.9) years before LIFE study enrollment and most often treated with surgery (*n* = 267). Compared with participants who did not report a history of cancer at baseline, participants with a history of cancer were older (79.7 vs 78.6 years; *P* < .001), less likely to be female (57.9 vs 69.8%; *P* < .001), less likely to be Black (11.3 vs 19.4%; *P* < .001), have a lower BMI (29.5 vs 30.4 kg/m^2^; *P* = .010), and engage in less moderate-intensity physical activity (184.9 vs 197.8 minutes per week; *P* = .019) at baseline. Compared with participants who did not report a history of cancer, cancer survivors had similar SPPB scores (7.4 vs 7.4 points; *P* = .60) and 400-m walk speed (0.81 vs 0.82 m/s; *P* = .20) at baseline.

**Table 1.** Baseline characteristics of the participants.

Characteristic	Cancer Survivor Subgroup (*n* = 371)		Non-Cancer Survivor Subgroup (*n* = 1264)	
	Physical activity (*n* = 181)	Health education (*n* = 190)	Physical activity (N = 637)	Health education (*n* = 627)
Age, y	79.7 (5.1)	79.8 (5.5)	78.4 (5.2)	78.8 (5.1)
Sex, No. (%)				
Male	72 (39.8%)	84 (44.2%)	199 (31.2)	182 (29.0%)
Female	109 (60.2%)	106 (55.8%)	438 (68.8)	445 (71.0%)
Race, No. (%)				
White	150 (82.9%)	158 (83.2%)	454 (71.3%)	477 (76.1%)
Black	23 (12.7%)	19 (10.0%)	140 (22.0%)	106 (16.9%)
Other	8 (4.4%)	13 (6.8)	43 (6.7%)	44 (7.0%)
Ethnicity, No. (%)				
Non-Hispanic	174 (96.1%)	182 (95.8%)	608 (95.4%)	603 (96.2%)
Hispanic	6 (3.3%)	8 (4.2%)	25 (3.9%)	22 (3.5%)
Unknown	1 (0.5%)	0 (0.0%)	4 (0.6%)	2 (0.3%)
SPPB score, 0-12				
Continuous	7.4 (1.6)	7.2 (1.6)	7.3 (1.6)	7.4 (1.6)
<8 (mobility disability) No. (%)	77 (42.5%)	92 (48.4%)	276 (43.3%)	286 (45.6%)
400m walking speed, m/s	0.83 (0.17)	0.79 (0.17)	0.82 (0.16)	0.82 (0.16)
BMI, kg/m^2^	29.3 (5.5)	29.7 (5.8)	30.3 (5.7)	30.5 (6.3)
Accelerometry of moderate physical activity, min/week	184.1 (164.8)	176.8 (164.5)	193.4 (157.5)	197.0 (182.1)
Cancer history, No. (%)				
Breast	57 (31.5%)	57 (30.0%)	—	—
Prostate	43 (23.7%)	51 (26.8%)	—	—
Colorectal	15 (8.3%)	23 (12.1%)	—	—
Uterine/endometrial	8 (4.4%)	8 (4.2%)	—	—
Lung	6 (3.3%)	5 (2.6%)	—	—
Other	73 (40.3%)	73 (38.4%)	—	—
Time since cancer diagnosis, y	12.3 (10.7)	11.6 (9.0)	—	—
Cancer treatment history, No. (%)				
Surgery	125 (69.1%)	142 (74.7%)	—	—
Radiotherapy	79 (43.6%)	74 (38.9%)	—	—
Chemotherapy	40 (22.1%)	36 (18.9%)	—	—
Hormonal therapy	22 (12.1%)	21 (11.0%)	—	—
Other	22 (12.1%)	11 (5.8%)	—	—

A total of 371 unique patients reported a total of 419 cancers; 330 patients reported a history of 1 cancer, 35 patients reported a history of 2 cancers, 5 patients reported a history of 3 cancers, and 1 participant reported a history of 4 cancers. Values are mean (SD) unless otherwise noted.

The median attendance to the health education group sessions was 82.8% (interquartile range, 64.1%-90.4%) in participants without a history of cancer and 80.8% (interquartile range, 58.7%-89.2%) in participants with a history of cancer (*P* = .12). The median attendance to the physical activity group sessions was 71.3% (interquartile range, 50.0%-83.5%) in participants without a history of cancer and 71.6% (interquartile range, 48.4%-82.6%) in participants with a history of cancer (*P* = .97). Compared with the health education group, participants randomized to the physical activity group increased their objectively measured moderate-intensity physical activity [*P* < .001; Figure S2], but this was not modified by cancer history (*P* = .80).

**Figure 1.**
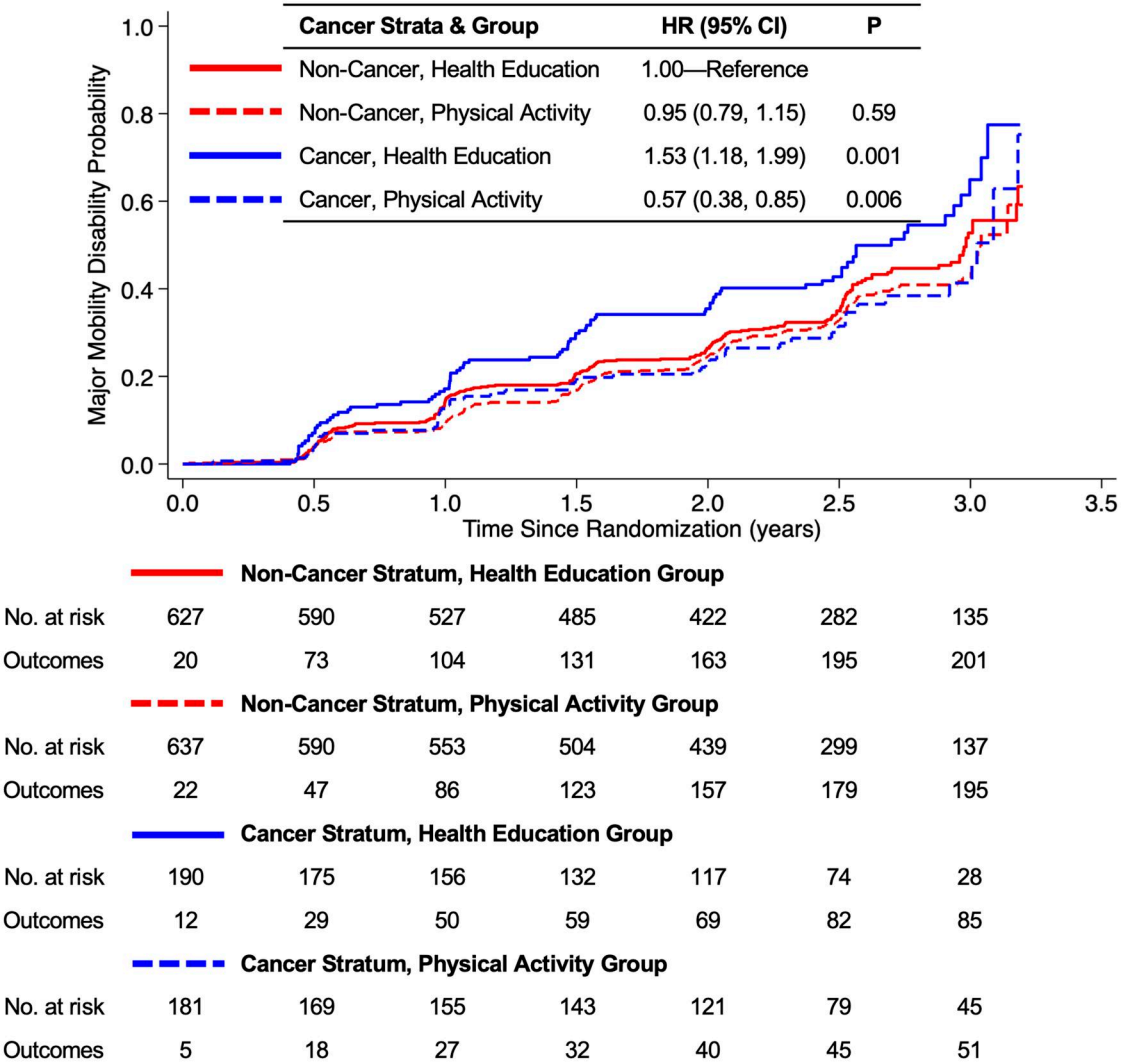
Time to major mobility disability by cancer history at enrollment and randomized group.

During a median follow-up time of 2.6 years (interquartile range, 2.3-3.1 years), major mobility disability occurred in 536 participants. A history of cancer modified the effect of the randomized group on major mobility disability (*P* = .006; Table 2; Figure 1). Among those randomized to the health education program, participants with a history of cancer were 53% more likely to develop major mobility disability compared with participants who did not have a history of cancer (HR = 1.53; 95% CI = 1.18 to 1.99; *P* = .001). Among participants with a history of cancer, those randomized to the physical activity program were 43% less likely to develop major mobility disability compared with the health education program (HR = 0.57; 95% CI = 0.40 to 0.82; *P* = .003). Conclusions of the sensitivity analysis were consistent with the main analysis (Table S1). Among the 3 most common types of cancer (breast, prostate, and colorectal), cancer type did not modify the effect of the randomized group on major mobility disability (*P* = .95), nor did the type of cancer treatment received (*P* = .14).

**Table 2.** Effect modification of randomized group by cancer history at enrollment on major mobility disability and persistent mobility disability.

	Health education group		Physical activity group		HR (95% CI) for randomized group within cancer survivor strata
	No. with event / No. at risk	HR (95% CI)	No. with event / No. at risk	HR (95% CI)	
Mobility disability					
Non-Cancer Survivor	204 / 627	1.00—Reference	195 / 637	0.95 (0.79 to 1.15); *P* = .59	0.95 (0.78 to 1.16); *P* = .63
Cancer Survivor	86 / 190	1.53 (1.18 to 1.99); *P* = .001	51 / 181	0.57 (0.38 to 0.85); *P* = .006	0.57 (0.40 to 0.82); *P* = .003
Persistent mobility disability					
Non-Cancer Survivor	119 / 627	1.00—Reference	101 / 637	0.83 (0.64 to 1.09); *P* = .18	0.84 (0.64 to 1.09); *P* = .19
Cancer Survivor	50 / 190	1.47 (1.05 to 2.05); *P* = .026	24 / 181	0.55 (0.31 to 0.96); *P* = .036	0.50 (0.30 to 0.82); *P* = .006

Estimates are adjusted for performance site and sex (both used to stratify randomization).

**Figure 2.**
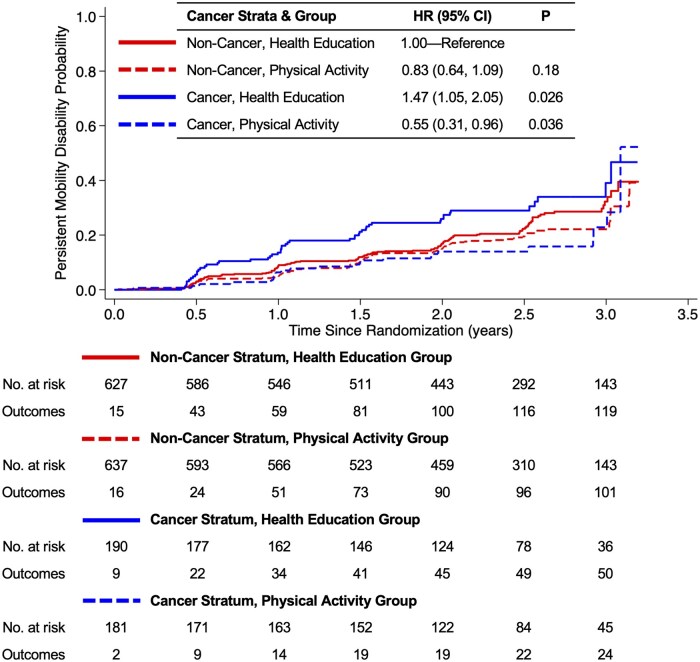
Time to persistent mobility disability by cancer history at enrollment and randomized group.

Persistent mobility disability occurred in 294 participants. A history of cancer modified the effect of the randomized group on persistent mobility disability (*P* = .036; Table 2; Figure 2). Among those randomized to the health education program, participants with a history of cancer were 47% more likely to develop persistent mobility disability compared with participants who did not have a history of cancer (HR = 1.47; 95% CI = 1.05 to 2.05; *P* = .026). Among participants with a history of cancer, those randomized to the physical activity program were 50% less likely to develop persistent mobility disability compared with the health education program (HR = 0.50; 95% CI = 0.30 to 0.82; *P* = .006). Conclusions of the sensitivity analysis were consistent with the main analysis. Among the 3 most common types of cancer (breast, prostate, and colorectal), cancer type did not modify the effect of the randomized group on persistent mobility disability (*P* = .42), nor did the type of cancer treatment received (*P* = .27).

Adverse events in the overall population by randomized group have been reported.^10^ A history of cancer did not statistically significantly modify the effect of the randomized group on adverse events (Table S2), including muscle or joint ache (*P* = .73), muscle or joint stiffness (*P* = .68), falls (*P* = .22), dizziness (*P* = .34), emergency room utilization (*P* = .071), inpatient hospitalization (*P* = .096), or death (*P* = .59).

## Discussion

This secondary analysis of the LIFE trial demonstrates that cancer survivors have an increased risk of mobility disability compared with non-cancer controls, and physical activity may attenuate this risk. Among those randomized to the health education (control) program, participants with a history of cancer were 53% more likely to develop major mobility disability compared with participants who did not have a history of cancer; this effect could be as small as 18% and as large as 99%. Participants with a history of cancer who were randomized to the physical activity (intervention) program were 43% less likely to develop major mobility disability compared with participants who did not have a history of cancer and who were randomized to health education; this effect could be as small as 18% and as large as 60%. This analysis offers at least 2 noteworthy observations.

First, in the absence of preventive intervention, cancer survivors are at a higher risk of developing mobility disability than individuals without a history of cancer. This analysis provides robust data to support the hypothesis that cancer survivors are susceptible to an accelerated aging phenotype, creating a vulnerability to develop geriatric syndromes such as frailty, sarcopenia, comorbidity burden, and mobility disability.^24^^,^^25^ In the LIFE trial, 45.3% of cancer survivors randomized to the health education program developed objectively defined major mobility disability during the follow-up period. This result is similar to a population-based survey where 39.7% of cancer survivors self-reported the inability to walk 400 m (eg, major mobility disability).^26^ Self-reported mobility disability in cancer survivors is associated with a 2-fold increased risk of all-cause and cancer-specific mortality.^27^

Second, participation in physical activity may reduce the risk of major mobility disability in cancer survivors. Among cancer survivors in the LIFE trial, the absolute risk difference between the health education and physical activity programs for the major mobility disability endpoint was 17.1% (95% CI = 7.4% to 26.7%). In addition, the physical activity group had a lower risk of persistent mobility disability compared with the health education group, indicating that physical activity may reverse mobility disability after it has occurred. Among cancer survivors in the LIFE trial, the absolute risk difference between the health education and physical activity programs for the persistent mobility disability endpoint was 13.1% (95% CI = 5.1% to 21.0%).

Compared with the health education program, the physical activity program led to meaningful increases in objectively measured moderate-intensity physical activity for cancer survivors (between-group difference at 0.5-, 1-, and 2-years of 51.5, 48.6, and 31.2 minutes per week, respectively (all *P* < .001)). Among cancer survivors, the physical activity program did not appear to increase the risk of adverse events, such as musculoskeletal events, falls, dizziness, emergency room utilization, hospitalization, and death compared with the health education program, although some of these comparisons may have limited statistical power. This analysis extends what is known about the benefits of physical activity in cancer survivors.^28^

An individual patient data meta-analysis of 32 randomized trials that included nearly 4000 cancer survivors (mean (SD) age = 54.5 (11.5) years) reported that randomization to exercise vs control caused a small but statistically significant increase in self-reported physical functioning (standardized mean difference = 0.32; 95% CI = 0.20 to 0.44).^29^ Moreover, in this individual patient data meta-analysis, patients with low physical function at study enrollment reported the largest magnitude of benefit from exercise training.^30^ However, blinding participants to randomized group assignments in exercise and physical activity trials is methodologically challenging, and the degree to which patient-reported endpoints of small treatment effects can be reliably interpreted may be difficult.^31^

There are limitations to this analysis and the LIFE trial. The principal limitation of this analysis is that the hypothesis of effect modification by cancer history was not prespecified in the study protocol. Consequently, this unplanned analysis should be considered hypothesis-generating but provides a robust preliminary assessment of intervention efficacy to inform the design of larger definitive studies in cancer survivors at unique points in the cancer care continuum. Although participants who reported a history of cancer at trial enrollment were required to have cancer without distant metastases and have completed cancer-directed therapy, we had limited information on the specific cancer staging, which may oversimplify heterogeneity in treatment effects. Cancer history was self-reported and, therefore, vulnerable to recall bias. The participants who reported a history of cancer were often long-term survivors of cancer, and the generalizability of these findings to patients more proximal to cancer diagnosis is uncertain. This analysis also did not have specific treatment information beyond knowing what modalities were utilized for cancer therapy. The heterogeneity of cancers enrolled limited our ability to conduct informative subgroup analyses due to the small number of major mobility disability events within a specific cancer type. Lastly, all participants in this study were self-selected, and the extent to which these findings can be generalized to the broader population of cancer survivors is unknown.

This analysis and the LIFE trial have important strengths. The participants in this study were all aged 70-89 years with mobility limitations. This population is typically underrepresented in oncology clinical trials and fills an important knowledge gap in cancer care.^32^ Participants were enrolled from a blend of urban, suburban, and rural centers, which broadly enhances the generalizability of these findings. The intervention was designed for scalability, requiring no specialized equipment, and led to meaningful increases in objectively measured moderate-intensity physical activity. The primary endpoint of time to major mobility disability is relevant to public health and medicine because the ability to walk without assistance is a key determinant for independent functioning and maintaining a high quality of life, and preserving the ability to walk 400 m is a surrogate for the ability to ambulate independently in the community.^33^^,^^34^

Cancer survivors had an increased risk of mobility disability compared with non-cancer controls, and physical activity was associated with an attenuation of this risk in this secondary analysis of the LIFE randomized trial. These observations provide robust foundational data to support the premise that interventions that prevent functional decline may extend the health span of cancer survivors.^9^ Further studies are needed to confirm these novel findings.

## Supplementary Material

pkaf084_Supplementary_Data

## Data Availability

Data are available at the NIA AgingResearchBiobank (https://agingresearchbiobank.nia.nih.gov).

## References

[1] Tonorezos E , Devasia T, Mariotto AB, et al Prevalence of cancer survivors in the United States. J Natl Cancer Inst. 2024;116:1784-1790. 39002121 10.1093/jnci/djae135 PMC11542986

[2] Burg MA , Adorno G, Lopez ED, et al Current unmet needs of cancer survivors: analysis of open-ended responses to the American Cancer Society Study of Cancer Survivors II. Cancer. 2015;121:623-630. 25581252 10.1002/cncr.28951

[3] Stein KD , Syrjala KL, Andrykowski MA. Physical and psychological long-term and late effects of cancer. Cancer. 2008;112:2577-2592. 18428205 10.1002/cncr.23448 PMC7047657

[4] Jette AM. Toward a common language for function, disability, and health. Phys Ther. 2006;86:726-734. 16649895

[5] Cespedes Feliciano EM , Vasan S, Luo J, et al Long-term trajectories of physical function decline in women with and without cancer. JAMA Oncol. 2023;9:395-403. 36656572 10.1001/jamaoncol.2022.6881 PMC9857739

[6] Petrick JL , Reeve BB, Kucharska-Newton AM, et al Functional status declines among cancer survivors: trajectory and contributing factors. J Geriatr Oncol. 2014;5:359-367. 24981125 10.1016/j.jgo.2014.06.002 PMC4254190

[7] Cao C , Yang L, Schmitz KH, et al Prevalence and cancer-specific patterns of functional disability among US cancer survivors, 2017-2022. J Clin Oncol. 2024;42:2257-2270. 38574313 10.1200/JCO.23.02536 PMC11470834

[8] Gonzalo-Encabo P , Vasbinder A, Bea JW, et al Low physical function following cancer diagnosis is associated with higher mortality risk in postmenopausal women. J Natl Cancer Inst. 2024;116:1035-1042. 38449287 10.1093/jnci/djae055 PMC11223816

[9] Rowland JH , Bellizzi KM. Cancer survivorship issues: life after treatment and implications for an aging population. J Clin Oncol. 2014;32:2662-2668. 25071099 10.1200/JCO.2014.55.8361 PMC4164810

[10] Pahor M , Guralnik JM, Ambrosius WT, et al Effect of structured physical activity on prevention of major mobility disability in older adults: the LIFE study randomized clinical trial. JAMA. 2014;311:2387-2396. 24866862 10.1001/jama.2014.5616 PMC4266388

[11] Brown JC. Measures of physical function clarify the prognostic blur of cancer survivorship. J Natl Cancer Inst. 2024;116:999-1001. 38630585 10.1093/jnci/djae076 PMC11223869

[12] Fielding RA , Rejeski WJ, Blair S, et al The lifestyle interventions and independence for elders study: design and methods. J Gerontol A Biol Sci Med Sci. 2011;66:1226-1237. 21825283 10.1093/gerona/glr123 PMC3193523

[13] Stewart AL , Mills KM, King AC, et al CHAMPS physical activity questionnaire for older adults: outcomes for interventions. Med Sci Sports Exerc. 2001;33:1126-1141. 11445760 10.1097/00005768-200107000-00010

[14] Guralnik JM , Ferrucci L, Simonsick EM, et al Lower-extremity function in persons over the age of 70 years as a predictor of subsequent disability. N Engl J Med. 1995;332:556-561. 7838189 10.1056/NEJM199503023320902 PMC9828188

[15] Teng EL , Chui HC. The Modified Mini-Mental State (3MS) examination. J Clin Psychiatry. 1987;48:314-318. 3611032

[16] Matts JP , Lachin JM. Properties of permuted-block randomization in clinical trials. Control Clin Trials. 1988;9:327-344. 3203524 10.1016/0197-2456(88)90047-5

[17] Rejeski WJ , Axtell R, Fielding R, et al Promoting physical activity for elders with compromised function: the Lifestyle Interventions and Independence for Elders (LIFE) study physical activity intervention. Clin Interv Aging. 2013;8:1119-1131. 24049442 10.2147/CIA.S49737 PMC3775623

[18] Hoxie RE , Rubenstein LZ. Are older pedestrians allowed enough time to cross intersections safely? J Am Geriatr Soc. 1994;42:241-244. 8120306 10.1111/j.1532-5415.1994.tb01745.x

[19] Pahor M , Guralnik JM, Anton SD, et al Impact and lessons from the Lifestyle Interventions and Independence for Elders (LIFE) clinical trials of physical activity to prevent mobility disability. J Am Geriatr Soc. 2020;68:872-881. 32105353 10.1111/jgs.16365 PMC7187344

[20] Guralnik JM , Simonsick EM, Ferrucci L, et al A short physical performance battery assessing lower extremity function: association with self-reported disability and prediction of mortality and nursing home admission. J Gerontol. 1994;49:M85-M94. 8126356 10.1093/geronj/49.2.m85

[21] Troiano RP , Berrigan D, Dodd KW, et al Physical activity in the United States measured by accelerometer. Med Sci Sports Exerc. 2008;40:181-188. 18091006 10.1249/mss.0b013e31815a51b3

[22] Freedson PS , Melanson E, Sirard J. Calibration of the Computer Science and Applications, Inc. accelerometer. Med Sci Sports Exerc. 1998;30:777-781. 9588623 10.1097/00005768-199805000-00021

[23] Marsh AP , Lovato LC, Glynn NW, et al Lifestyle Interventions and Independence for Elders study: recruitment and baseline characteristics. J Gerontol A Biol Sci Med Sci. 2013;68:1549-1558. 23716501 10.1093/gerona/glt064 PMC3814232

[24] Guida JL , Ahles TA, Belsky D, et al Measuring aging and identifying aging phenotypes in cancer survivors. J Natl Cancer Inst. 2019;111:1245-1254. 31321426 10.1093/jnci/djz136 PMC7962788

[25] Guida JL , Agurs-Collins T, Ahles TA, et al Strategies to prevent or remediate cancer and treatment-related aging. J Natl Cancer Inst. 2021;113:112-122. 32348501 10.1093/jnci/djaa060 PMC7850536

[26] Schootman M , Aft R, Jeffe DB. An evaluation of lower-body functional limitations among long-term survivors of 11 different types of cancers. Cancer. 2009;115:5329-5338. 19676109 10.1002/cncr.24606 PMC2791371

[27] Brown JC , Harhay MO, Harhay MN. Self-reported major mobility disability and mortality among cancer survivors. J Geriatr Oncol. 2018;9:459-463. 29550343 10.1016/j.jgo.2018.03.004 PMC6113100

[28] Fuller JT , Hartland MC, Maloney LT, et al Therapeutic effects of aerobic and resistance exercises for cancer survivors: a systematic review of meta-analyses of clinical trials. Br J Sports Med. 2018;52:1311. 29549149 10.1136/bjsports-2017-098285

[29] Buffart LM , Kalter J, Sweegers MG, et al Effects and moderators of exercise on quality of life and physical function in patients with cancer: an individual patient data meta-analysis of 34 RCTs. Cancer Treat Rev. 2017;52:91-104. 28006694 10.1016/j.ctrv.2016.11.010

[30] Buffart LM , Sweegers MG, May AM, et al Targeting exercise interventions to patients with cancer in need: an individual patient data meta-analysis. J Natl Cancer Inst. 2018;110:1190-1200. 30299508 10.1093/jnci/djy161 PMC6454466

[31] US Food and Drug , Administration. Patient-reported outcome measures: use in medical product development to support labeling claims. Guidance for Industry. 2009. 10.1186/1477-7525-4-79 PMC1629006 17034633

[32] Sedrak MS , Freedman RA, Cohen HJ, et al Older adult participation in cancer clinical trials: a systematic review of barriers and interventions. CA Cancer J Clin. 2021;71:78-92. 33002206 10.3322/caac.21638 PMC7854940

[33] Buchner DM. One lap around the track: the standard for mobility disability? J Gerontol A Biol Sci Med Sci. 2008;63:586-587. 18559632 10.1093/gerona/63.6.586

[34] Fielding RA , LeBrasseurNK. Editorial: outcomes for regulatory approval in geriatrics: embracing loss of mobility and mobility disability as clinically meaningful therapeutic indications. J Nutr Health Aging. 2023;27:496-497.37498095 10.1007/s12603-023-1944-7

