# Supplementary material for: Physical activity and mobility disability in older adult cancer survivors
Source: JNCI Cancer Spectr. 2025 Sep 10;9(5):pkaf084. doi: 10.1093/jncics/pkaf084 (PMC12422780; doi:10.1093/jncics/pkaf084)
Supplement: pkaf084_Supplementary_Data [file pkaf084_supplementary_data.zip › Supplementary Material (FINAL).docx]

**Supplementary Material**

**Physical Activity and Mobility Disability in Older Adult Cancer Survivors**

Justin C. Brown, Ph.D.^1^ and Shengping Yang, Ph.D.^2^

^1^AdventHealth, 301 E Princeton St, Orlando, FL 32804; ^2^Pennington Biomedical Research Center, 6400 Perkins Rd, Baton Rouge, LA 70808, USA.

**Corresponding Author**

Justin C. Brown, Ph.D.

6400 Perkins Road

Baton Rouge, LA 70808

Phone: 225-763-2715

Email: Justin.Brown@pbrc.edu

**Short Title**

Physical Activity and Mobility Disability in Cancer Survivors

**Figure S1.** CONSORT diagram of participant flow through the study

**
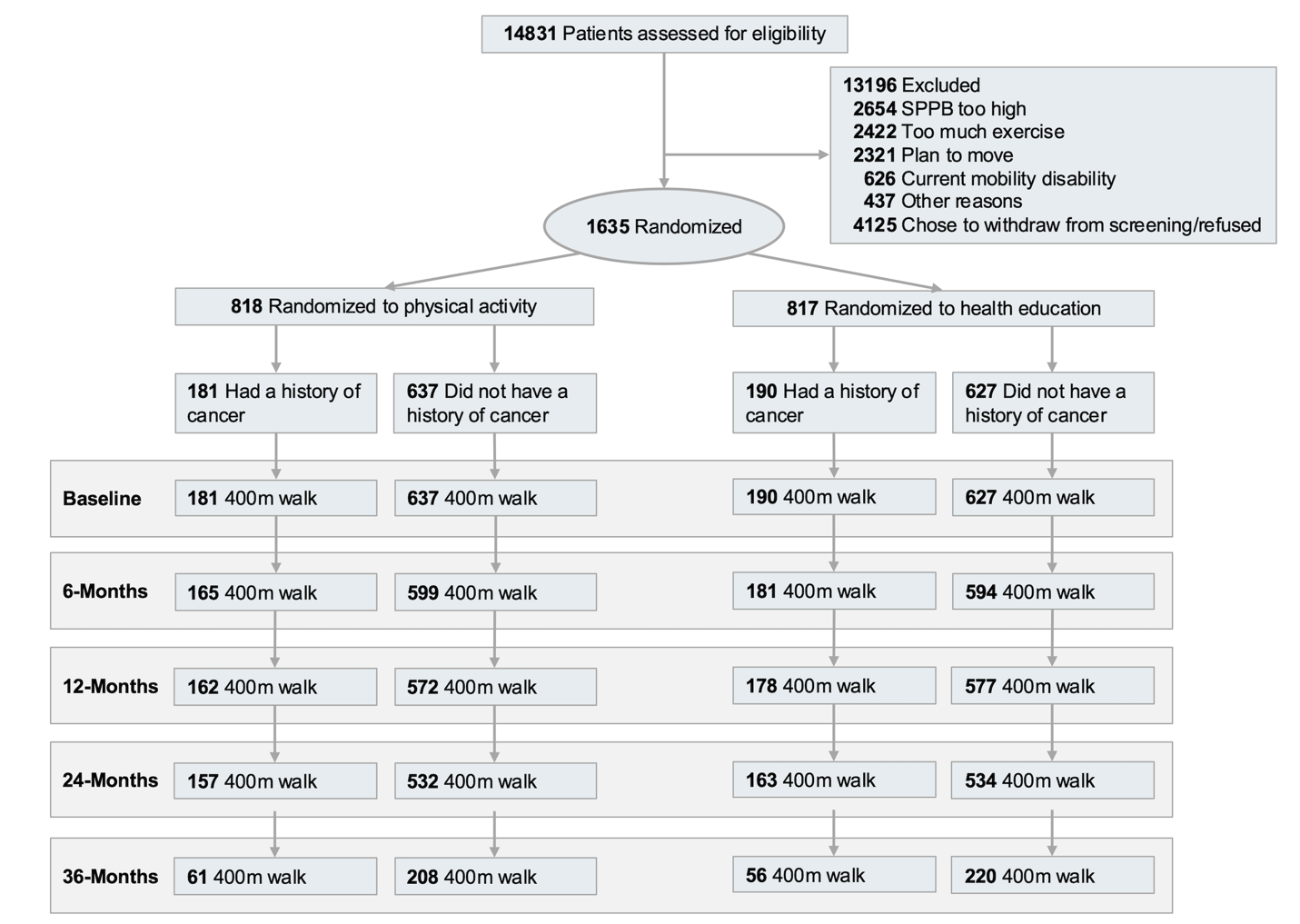
**

**Figure S2.** Accelerometry-Derived Physical Activity by Cancer History at Enrollment and Randomized Group

**
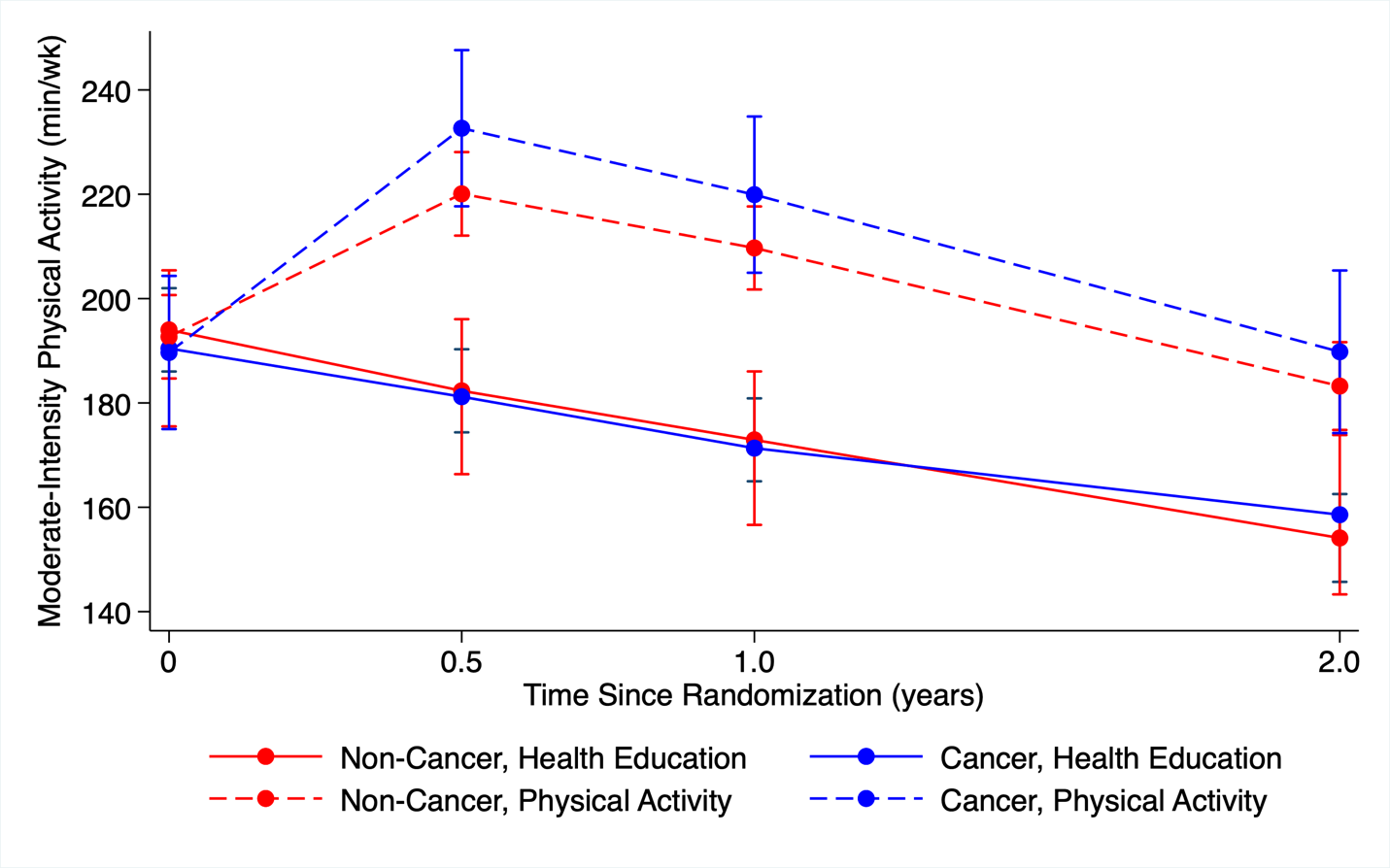
**

Effect modification by cancer history *P* = 0.80

Group-by-Time *P* < 0.001

**Table S1.** Effect Modification of Randomized Group by Cancer History at Enrollment on Major Mobility Disability and Persistent Mobility Disability

|  | **Health Education Group** | |  | **Physical Activity Group** | |  | **HR (95% CI) for Randomized Group within Cancer Survivor Strata** |
| --- | --- | --- | --- | --- | --- | --- | --- |
|  | **N with event / N at risk** | **Hazard Ratio (95% CI)** |  | **N with event / N at risk** | **Hazard Ratio (95% CI)** |  |  |
| **Mobility Disability** |  |  |  |  |  |  |  |
| Non-Cancer Survivor | 204 / 627 | 1.00⎯Reference |  | 195 / 637 | 0.96 (0.78, 1.17)  P=0.66 |  | 0.96 (0.78, 1.17)  P=0.67 |
| Cancer Survivor | 86 / 190 | 1.51 (1.16, 1.95)  P=0.002 |  | 51 / 181 | 0.57 (0.38, 0.86)  P=0.007 |  | 0.61 (0.42, 0.87)  P=0.007 |
| **Persistent Mobility Disability** |  |  |  |  |  |  |  |
| Non-Cancer Survivor | 119 / 627 | 1.00⎯Reference |  | 101 / 637 | 0.85 (0.65, 1.11)  P=0.25 |  | 0.86 (0.65, 1.12)  P=0.26 |
| Cancer Survivor | 50 / 190 | 1.44 (1.03, 2.02)  P=0.032 |  | 24 / 181 | 0.56 (0.32, 0.98)  P=0.041 |  | 0.53 (0.32, 0.87)  P=0.012 |

Estimates are adjusted for performance site and sex (both used to stratify randomization), and baseline characteristics that differed between participants with a history of cancer and those who did not report a history of cancer, including age, race, body mass index, and physical activity volume.

**Table S2.** Effect Modification of Randomized Group by Cancer History at Enrollment on Adverse Events

|  | **Health Education Group** | |  | **Physical Activity Group** | |  | **Relative Risk (95% CI) for Randomized Group within Cancer Survivor Strata** |
| --- | --- | --- | --- | --- | --- | --- | --- |
|  | **N with event / N at risk** | **Relative Risk (95% CI)** |  | **N with event / N at risk** | **Relative Risk (95% CI)** |  |  |
| **Muscle or joint ache^a^** |  |  |  |  |  |  |  |
| Non-Cancer Survivor | 29 / 627 | 1.00⎯Reference |  | 25 / 637 | 0.86 (0.50, 1.45)  P=0.56 |  | 0.87 (0.51, 1.46)  P=0.59 |
| Cancer Survivor | 10 / 190 | 1.09 (0.53, 2.28)  P=0.81 |  | 6 / 181 | 0.83 (0.28, 2.42)  P=0.73 |  | 0.72 (0.28, 1.89)  P=0.51 |
| **Muscle or joint stiffness^a^** |  |  |  |  |  |  |  |
| Non-Cancer Survivor | 24 / 627 | 1.00⎯Reference |  | 21 / 637 | 0.88 (0.49, 1.57)  P=0.67 |  | 0.90 (0.50, 1.60)  P=0.72 |
| Cancer Survivor | 8 / 190 | 1.04 (0.46, 2.35)  P=0.91 |  | 4 / 181 | 0.76 (0.21, 2.77)  P=0.68 |  | 0.74 (0.23, 2.38)  P=0.61 |
| **Falls^a^** |  |  |  |  |  |  |  |
| Non-Cancer Survivor | 53 / 627 | 1.00⎯Reference |  | 32 / 637 | 0.56 (0.37, 0.85)  P=0.007 |  | 0.57 (0.38, 0.87)  P=0.008 |
| Cancer Survivor | 13 / 190 | 0.79 (0.43, 1.45)  P=0.45 |  | 13 / 181 | 1.70 (0.73, 3.96)  P=0.22 |  | 1.04 (0.50, 2.18)  P=0.91 |
| **Dizziness^a^** |  |  |  |  |  |  |  |
| Non-Cancer Survivor | 8 / 627 | 1.00⎯Reference |  | 15 / 637 | 1.50 (0.65, 3.48)  P=0.33 |  | 1.53 (0.69, 3.42)  P=0.29 |
| Cancer Survivor | 6 / 190 | 1.71 (0.62, 4.72)  P=0.30 |  | 3 / 181 | 0.46 (0.10, 2.22)  P=0.34 |  | 0.76 (0.21, 2.92)  P=0.70 |
| **Emergency Room Utilization^b^** |  |  |  |  |  |  |  |
| Non-Cancer Survivor | 150 / 627 | 1.00⎯Reference |  | 204 / 637 | 1.33 (1.12, 1.60)  P=0.001 |  | 1.34 (1.12, 1.61)  P=0.001 |
| Cancer Survivor | 68 / 190 | 1.51 (1.19, 1.91)  P=0.001 |  | 65 / 181 | 0.74 (0.54, 1.03)  P=0.071 |  | 1.02 (0.78, 1.35)  P=0.871 |
| **Hospitalization^b^** |  |  |  |  |  |  |  |
| Non-Cancer Survivor | 273 / 627 | 1.00⎯Reference |  | 319 / 637 | 1.15 (1.02, 1.29)  P=0.018 |  | 1.15 (1.03, 1.30)  P=0.017 |
| Cancer Survivor | 106 / 190 | 1.31 (1.12, 1.53)  P=0.001 |  | 99 / 181 | 0.83 (0.67, 1.03)  P=0.096 |  | 0.95 (0.79, 1.15)  P=0.62 |
| **Death^b^** |  |  |  |  |  |  |  |
| Non-Cancer Survivor | 30 / 627 | 1.00⎯Reference |  | 33 / 637 | 1.06 (0.65, 1.71)  P=0.82 |  | 1.04 (0.64, 1.68)  P=0.87 |
| Cancer Survivor | 12 / 190 | 1.18 (0.61, 2.26)  P=0.62 |  | 15 / 181 | 1.27 (0.53, 3.06)  P=0.59 |  | 1.31 (0.63, 2.71)  P=0.47 |

Estimates are adjusted for performance site and sex (both used to stratify randomization). ^a^Individual participants may have experienced more than one instance of these adverse events in a Poisson model. ^b^The first instance was counted as an event in a generalized linear model.
